# Supplementary material for: The causal relationship between the human gut microbiota and pyogenic arthritis: a Mendelian randomization study
Source: Front Cell Infect Microbiol. 2024 Nov 26;14:1452480. doi: 10.3389/fcimb.2024.1452480 (PMC11629706; doi:10.3389/fcimb.2024.1452480)
Supplement: Supplementary file 1 [file DataSheet1.zip › mendelian test/ebi-a-GCST90027592.csv_forest.pdf]

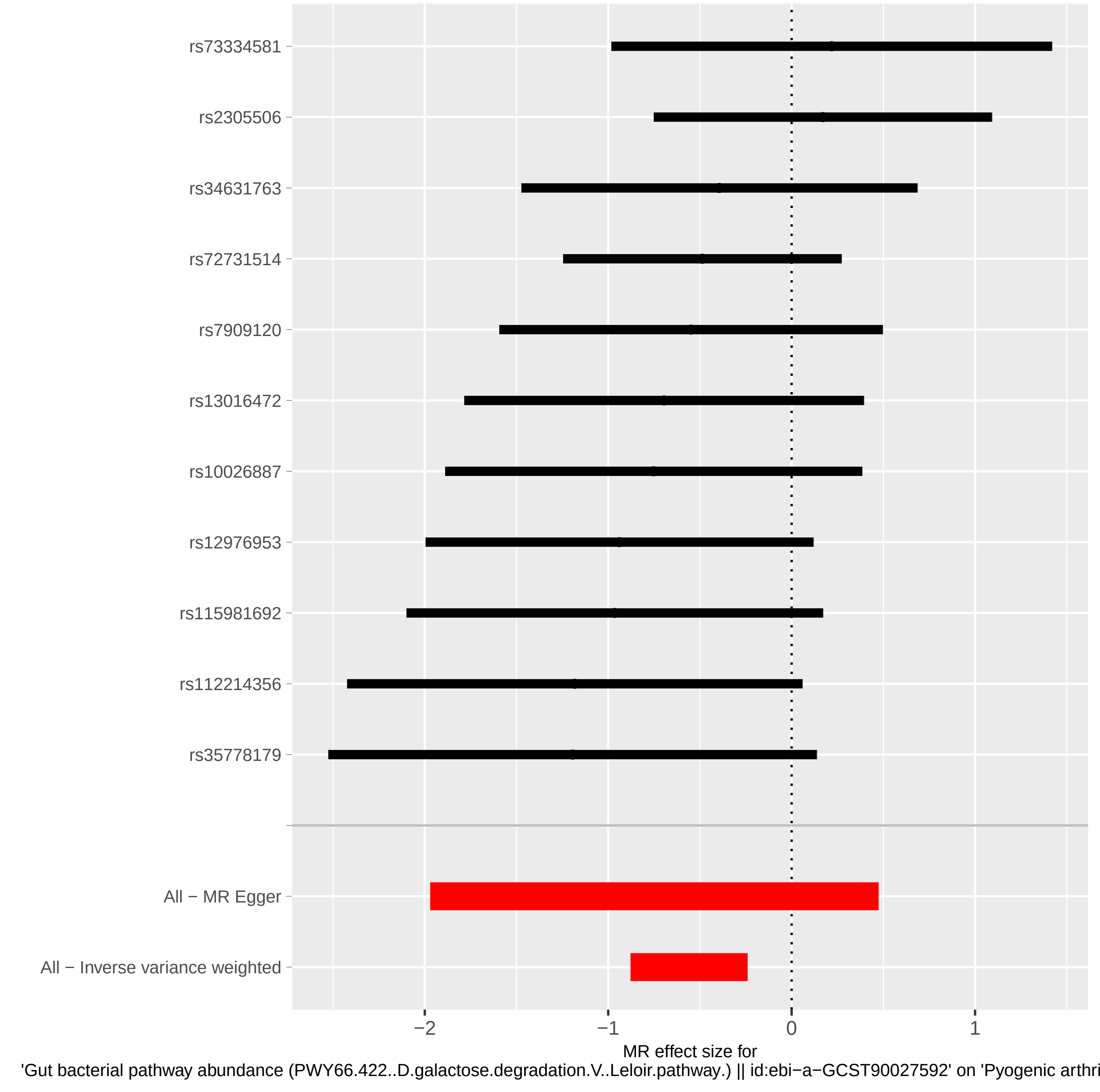
rs73334581
rs2305506
rs34631763
rs72731514
rs7909120
rs13016472
rs10026887
rs12976953
rs115981692
rs112214356
rs35778179
All − MR Egger
All − Inverse variance weighted
−2
−1
0
1
MR effect size for
'Gut bacterial pathway abundance (PWY66.422..D.galactose.degradation.V..Leloir.pathway.) || id:ebi−a−GCST90027592' on 'Pyogenic arthri
